# Determinants of Healthcare Use Based on the Andersen Model: A Systematic Review of Longitudinal Studies

**DOI:** 10.3390/healthcare9101354

**Published:** 2021-10-12

**Authors:** André Hajek, Benedikt Kretzler, Hans-Helmut König

**Affiliations:** Hamburg Center for Health Economics, Department of Health Economics and Health Services Research, University Medical Center Hamburg-Eppendorf, 20246 Hamburg, Germany; b.kretzler.ext@uke.de (B.K.); h.koenig@uke.de (H.-H.K.)

**Keywords:** healthcare use, health services research, Andersen, healthcare utilization, predisposing characteristics, enabling resources, need factors, systematic review, longitudinal studies, cohort studies, observational studies

## Abstract

The aim was to give an overview of longitudinal observational studies investigating the determinants of healthcare use explicitly using the Andersen model. To this end, three electronic databases (Medline, PsycINFO and CINAHL) were searched (and an additional hand search was performed). Longitudinal observational studies examining the determinants of healthcare use (outpatient physician services and hospital stays) based on the Andersen model were included, whereas disease-specific samples were excluded. Study quality was evaluated. The selection of studies, extraction of data and assessment of the studies were conducted by two reviewers. The following determinants of healthcare use were displayed based on the (extended) Andersen model: predisposing characteristics, enabling resources, need factors and psychosocial factors. In sum, *n* = 10 longitudinal studies have been included in our systematic review. The included studies particularly showed a longitudinal association between increased needs and higher healthcare use. Study quality was rather high. However, several studies did not conduct robustness checks or clarify the handling of missing data. In conclusion, this systematic review adds to our current understanding of the factors associated with healthcare use (mainly based on cross-sectional studies). It showed mixed evidence with regard to the association between predisposing characteristics, enabling resources and healthcare use longitudinally. In contrast, increased need factors (in particular, self-rated health and chronic conditions) were almost consistently associated with increased healthcare use. This knowledge may assist in managing healthcare use. Since most of the studies were conducted in North America or Europe, future longitudinal studies from other regions are urgently required.

## 1. Introduction

Healthcare use (HCU) mainly includes physician visits (particularly general practitioner (GP) and specialist visits) as well as hospitalization. One landmark theoretical model to study the determinants of HCU is called the Andersen Model [1] with the dimensions of predisposing characteristics (e.g., chronological age or sex), enabling resources (e.g., self-rated access to hospital or household income) and need factors (e.g., number of physical illnesses, mental health or self-rated health). Moreover, recent studies have suggested adding psychosocial factors such as loneliness and personality factors such as conscientiousness to the Andersen model [2].

To date, various cross-sectional studies—based on the Andersen model—exist to investigate the factors associated with HCU (e.g., [3,4]). A previous systematic review published almost ten years ago (i.e., in the year 2012) systematically synthesized studies analyzing the determinants of HCU based on the Andersen model [5]. While mixed determinants were included, an overall association between increased need factors and increased HCU has been found in a previous review [5]. However, in the past few years, several longitudinal studies have been published (for example, [6])—often demonstrating that increases in needs are associated with increases in HCU. Nevertheless, thus far, a systematic review systematically synthesizing longitudinal studies analyzing the determinants of HCU—explicitly using the Andersen model—is lacking. Consequently, the aim of this review was to close this gap in knowledge. In total, this knowledge may be beneficial to manage HCU and can help to avoid over-, under- and misuse (e.g., if particularly predisposing factors such as country of origin or sex are associated with HCU). Contrary, if need factors are exclusively associated with HCU based on a longitudinal approach, this could indicate that health services are used only if medically indicated.

Moreover, a systematic review solely based on longitudinal studies in this research area is required since longitudinal studies can assist in clarifying the directionality between the factors. Additionally, and in contrast to results based on cross-sectional studies, results based on longitudinal studies can produce consistent estimates when certain panel regression models were applied.

It should be noted that other domains of HCU exist, such as check-ups, dental services or mental HCU. Nevertheless, we concentrate on outpatient physician visits and hospitalization in this current systematic review due to homogeneity in the dependent variables. Furthermore, these outcome measures differ in their correlates.

## 2. Material and Methods

Our systematic review was performed in accordance with the Preferred Reporting Items for Systematic Reviews and Meta-Analysis guidelines [7]. Moreover, it is registered with the International Prospective Register of Systematic Reviews (PROSPERO, registration number: CRD42020193198). Additionally, a study protocol for our systematic review has recently been published [8]. All steps (study selection, data extracting and quality assessment) were independently conducted by two reviewers. When opinions differed between the reviewers, discussions were used to resolve it. A third party (H.H.K.) was used (if required).

### 2.1. Search Strategy and Eligibility Criteria

In May and June 2021, a systematic literature search was conducted (Medline, PsycINFO and CINAHL). In Table 1, the search query for Medline is presented.

Inclusion criteria were as follows: (i) longitudinal observational studies examining the determinants of HCU in terms of outpatient physician services (such as GP visits or specialist visits in total) or hospitalization, (ii) studies explicitly using the Andersen model, (iii) measurement of important variables with suitable tools, (iv) studies in German or English language and (v) studies published in peer-reviewed, scientific journals. In contrast, exclusion criteria were (i) studies exclusively based on samples with a specific disorder (e.g., individuals with mental disorders) and (ii) studies solely examining single medical specialties (other than GP visits) such as ophthalmologist (visits).

No restrictions were made regarding location or time of the studies. A pretest was conducted prior to final eligibility criteria (using a sample of 100 titles/abstracts). However, our eligibility criteria did not change.

### 2.2. Study Selection

The identified studies were imported into EndNote X8 (Clarivate Analytics, Philadelphia, PA, USA). Studies were evaluated for inclusion/exclusion based on a two-step approach starting with a title/abstract screening and a full-text screening afterwards. Furthermore, a hand search was conducted.

### 2.3. Data Extraction and Analysis

One reviewer (B.K.) performed extraction of the data and a second reviewer (A.H.) checked it. If required, the study authors were contacted for clarification. Data on study design, measurement of HCU, characteristics of the sample, size of the sample and key findings were extracted. The key findings are displayed based on the Andersen model.

### 2.4. Quality Assessment

As yet, there is no consensus regarding a quality assessment tool for HCU studies. In this study, we used a tool developed by Stuhldreher et al. [9] and refined by Hohls et al. [10]. For further details, please see Table 2 in a study conducted by Hajek et al. [11].

## 3. Results

### 3.1. Overview of Included Studies

**Figure 1:**
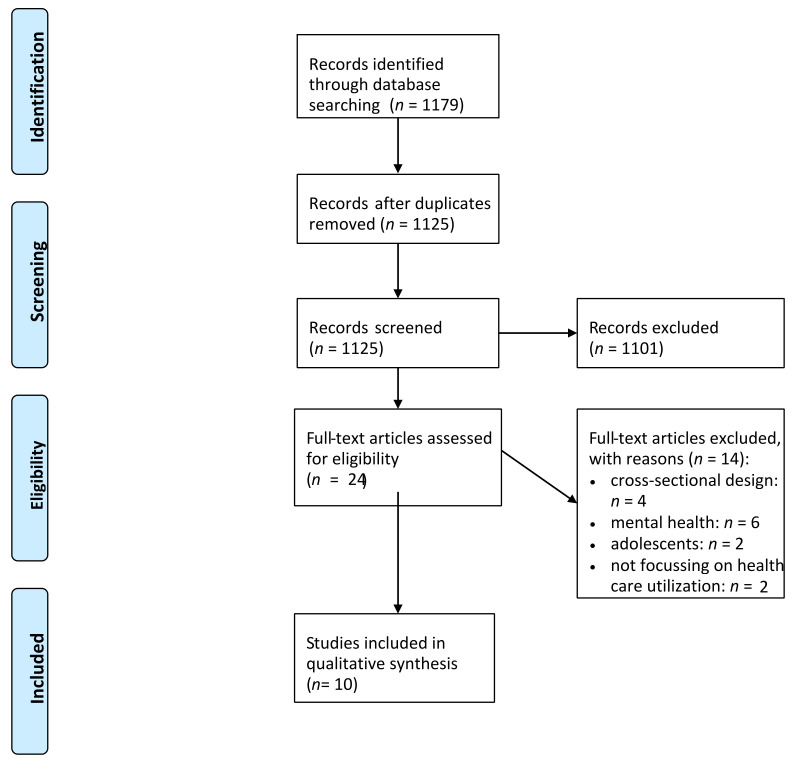
Flow chart. PRISMA 2009 Flow Diagram.

The study selection process is displayed in Figure 1.

In total, *n* = 10 studies are included in our review [12,13,14,15,16,17,18,19,20,21]. Important findings of the studies are shown in Table 2 (if shown, adjusted results are given in Table 2). Data came from North America (*n* = four, with three studies from the United States and one study from Canada), Europe (*n* = five, all from Germany) and Asia (*n* = one, South Korea). The observation period ranged from ten months to twelve years. The number of waves used ranged from two to nine waves.

While two studies only examined the frequency of physician visits, three studies exclusively examined the likelihood of hospitalization. Moreover, five studies used different outcomes (such as the frequency of GP as well as specialist visits and hospitalization). All studies investigated the outpatient physician visits/hospitalization in the last three to twelve months.

One study used a Cox proportional hazards model [13]. Furthermore, all five German studies used specifically designed panel regression models [15,16,17,18,19]. Three studies used a static set of baseline characteristics (such as sex) to predict subsequent outcomes [12,14,21] or used pooled models [20]—and, therefore, do not fully exploit the longitudinal data structure [22]. Furthermore, they may have produced inconsistent estimates due to unobserved heterogeneity [22].

Most of the studies focused on middle-aged or older individuals. The sample size ranged from 270 to 28,574 individuals and the proportion of women ranged from 30 to 69%. Additional details are given in Table 2.

We will display our key findings in the following upcoming sections according to the extended [2] Andersen model for reasons of clarity and readability: predisposing characteristics, enabling resources, need factors and psychosocial factors.

### 3.2. Predisposing Characteristics

In total, *n* = nine studies examined the association between predisposing characteristics and HCU longitudinally.

With regard to age, eight studies examined the association between age and HCU. Three studies did not find an association between these factors [17,18,19]. Moreover, while three studies found a positive association between these factors [12,13,16], two studies found negative associations [15,20].

With regard to sex, four studies examined the association between sex and HCU. While two studies found an association between being female and higher HCU [12,20], two other studies did not find a significant association between sex and HCU [13,17].

With regard to educational level, five studies examined an association between educational level and HCU [12,13,17,19,20]. However, none of the studies found a significant association between these factors.

With regard to marital status, eight studies investigated the association between marital status and HCU. While six studies did not identify an association between these factors [12,13,16,17,18,19], two studies found an association between being married or having a partner and increased HCU [15,20].

Other predisposing characteristics were only included occasionally. For example, three studies examined an association between employment status and HCU. While two studies found an association between unemployment and higher HCU [15,16], one study did not determine a significant association between these factors [19]. Moreover, three studies examined the association between race and HCU. Two studies did not identify an association between race and HCU [12,13], whereas one study showed that being African American (compared to others) was associated with a higher likelihood of hospitalization [21].

### 3.3. Enabling Resources

In total, all the *n* = 10 studies examined the association between enabling resources and HCU longitudinally.

With regard to income, four studies examined the association between income and HCU. None of these studies found a significant association [15,16,19,20]. Analogously, health insurance was not significantly associated with HCU in the three studies that examined such an association [13,15,21]. While the German study compared statutory health insurance and private health insurance with regard to HCU [15], one study conducted in the United States compared individuals who did not have private insurance in addition to Medicare and individuals with private insurance among older African American and Caucasian Medicare Beneficiaries [13], and another study compared homeless individuals with health insurance with homeless individuals without health insurance in the United States [21].

With regard to social support/social network, two out of the four studies did not find an association between social support/social network and HCU [18,21]. In contrast, one study found an association between higher social network and an increased likelihood of hospitalization among the oldest old [17]. Similar findings were made by Clay et al. [13].

### 3.4. Need Factors

In total, all the *n* = 10 studies examined the association between need factors and HCU longitudinally. In general, all the studies found an association between higher needs and higher HCU. In particular, strong associations were found between chronic conditions as well as low self-rated health and increased HCU [12,16,17,19,21]. However, two studies also found that disability/functional impairment was not associated with increased HCU [18,20].

With regard to mental health, three studies also found an association between decreased mental health and increased HCU [15,17,18], whereas one study did not find such an association among homeless individuals [21].

### 3.5. Psychosocial Factors

In total, *n* = one study examined the longitudinal association between psychosocial factors and HCU [19]. More precisely, this study examined the association between general locus of control and HCU. This study showed that while the external locus of control was positively associated with a higher frequency of physician visits (ß = 0.00, *p* < 0.05), the internal locus of control was not significantly associated with the frequency of physician visits longitudinally [19].

### 3.6. Quality Assessment

The quality assessment for our included studies is given in Table 3. In total, 87.5 to 93.8% of the criteria were achieved by the included studies. The categories that were commonly not fulfilled were the treatment of missing data (30% fulfilled) and conducting sensitivity analyses (50% fulfilled).

## 4. Discussion

The aim of this systematic review was to give an overview of longitudinal observational studies investigating the determinants of healthcare use explicitly using the Andersen model. This knowledge adds to our current understanding of the factors associated with HCU (mainly based on cross-sectional studies). These cross-sectional studies showed, in general, mixed evidence regarding the associations between predisposing characteristics, enabling resources and HCU [5]. Furthermore, cross-sectional studies mainly found an association between higher needs and higher HCU [5].

With regard to the association between predisposing characteristics and HCU, findings were rather mixed or inconclusive. More precisely, the findings were mixed regarding the association between age and HCU (also depending on the model specification). Furthermore, most of the studies did not identify an association between marital status as well as education and HCU. Moreover, there was inconclusive evidence regarding the association between sex, employment status as well as race and HCU. Due to the limited knowledge (mainly based on data taken from Germany or the United States), much more research based on longitudinal data is required to clarify whether the aforementioned predisposing characteristics are important for HCU in other regions.

With regard to the association between enabling resources and HCU, none of the studies found an association between income and HCU. However, it should be noted that all of the studies used data from Germany (and one study used data from South Korea). Thus, future research in other regions (e.g., United States) is urgently required since the German healthcare system (where enabling resources generally do not drive HCU [16]) may heavily contribute to these results.

Since only three studies compared very different groups of health-insured individuals (Germany or United States), future longitudinal studies in this area are required. Moreover, there is mixed evidence regarding the association between social support/social network and HCU. A positive association between these factors may be explained by the fact that the social network urges an individual to check their illness symptoms and can help with transportation.

With regard to the association between need factors and HCU, there was clear evidence for a positive association between these factors (i.e., higher needs are associated with higher HCU), particularly between chronic conditions as well as self-rated health and HCU. This is very plausible since an increased need reflects illness symptoms that can be checked by consulting a physician and has been shown by numerous cross-sectional studies [5]. Future longitudinal studies in this area are required to clarify the association between the onset of single diseases and HCU as well as between different multimorbidity patterns and HCU.

With regard to the association between psychosocial factors and HCU, only one study examined this association. This study showed an association between an external locus of control and increased HCU among the general adult population in Germany. Drawing general conclusions from it is, thus, difficult. As some cross-sectional studies showed associations between psychosocial factors as well as personality-related factors and HCU, future longitudinal studies are required to clarify the association between these factors.

The study quality only slightly varied between the studies and was generally high. It is likely that the high quality of the included studies can be explained by the publication date (i.e., seven out of the ten studies were published in the past five years). The most common shortcomings are that robustness checks (i.e., sensitivity analyses) were not conducted. However, conducting such checks is required to ensure that the study findings did not depend, e.g., on the model specification (e.g., which health-related factors are included) or the analytical approach (e.g., panel regression models vs. cross-sectional regression models) used. Consequently, such checks are recommended by current guidelines [23]. Moreover, most of the studies did not describe how they dealt with missing data. Hence, future research could tackle this issue, for example, by using a full-information maximum likelihood approach [24] since such approaches can produce more accurate findings [25].

The comparability of the included studies is somewhat restricted. While the majority of the studies used large, mostly representative samples [13,15,16,17,18,19,20], two studies used data from homeless individuals that are difficult to generalize [14,21]. Moreover, the studies differ in the time horizon. It should also be acknowledged that several of the studies included in our review used specific regression models for dealing with longitudinal data (which is important to receive consistent estimates [22]) [13,15,16,17,18,19], whereas some studies did not use such analytical approaches and are, therefore, prone to bias [12,13,20,21]. Moreover, all the studies included in our review only used self-reported HCU, which is prone to some recall bias [26]. If data are available, claim data should be linked to survey data. The existing studies used rather ‘classic’ factors included in the Andersen model such as age or chronic conditions. Future research regarding the association between psychosocial factors and HCU is, therefore, required. For example, factors such as loneliness, conscientiousness or neuroticism may moderate the association between need factors and HCU. Such factors could also be integrated in a revised version of the Andersen model, as recently suggested [2].

Furthermore, restrictions were not applied regarding the time and location of the studies. The location of the studies may introduce some heterogeneity in our findings, since the healthcare systems often differ between the countries. Moreover, as outlined above, eight of the ten studies included in our systematic review were published in the past ten years, whereas the two remaining studies were published in the 2000s. This may be explained by the increased availability of longitudinal secondary data. We assume that far more longitudinal studies on this topic will be published in the next few years.

Some strengths and limitations of our systematic review are worth highlighting. This is the first systematic review examining the determinants of HCU based on the Andersen model exclusively including longitudinal studies. This focus may ascertain a sufficient quality of the studies and may assist in making more valid conclusions. For example, by exploiting the longitudinal data structure, the problem of unobserved heterogeneity can be reduced [27], which is a main advantage in comparison to cross-sectional data. Additionally, using longitudinal data can assist in clarifying the directionality [27]. In particular, the included studies from Germany used such panel regression models and can, therefore, reduce the problem of unobserved heterogeneity. A quality assessment was performed. Two reviewers conducted important steps in this review. Due to study heterogeneity, a meta-analysis could not be conducted. While the restriction to peer-reviewed articles may exclude potentially suitable findings (e.g., grey literature), it ascertains a certain quality. Moreover, since we restricted our search to two languages, potentially suitable studies published in other languages may not be determined.

The Andersen model was initially developed in the 1960s. It was updated and refined over time. Most frequently, the 1995 version of the Andersen model was used in the included studies. However, for example, one study included in our systematic review explicitly focused on homeless individuals and, therefore, used the Gelberg–Andersen Behavioral Model for Vulnerable Populations [28] as a theoretical background. Most of the included studies used secondary data. Therefore, they were frequently restricted in the independent variables they could select from. The use of secondary data and the use of older versions may be reasons why the included studies often focused on similar variables (e.g., age, sex, income, or health-related factors). As also noted above, future studies should also look at the neglected factors of the Andersen model, e.g., financing at the contextual level such as available resources in the community for health services (e.g., rate of health insurance coverage or health care expenditures) or organizational factors such as physician density or office hours.

## 5. Conclusions

This systematic review adds to our current understanding of the factors associated with HCU (mainly based on cross-sectional studies). Similarly to previous cross-sectional studies [5], it showed mixed evidence with regard to the association between predisposing characteristics, enabling resources and HCU longitudinally. In contrast, need factors (in particular, self-rated health and chronic conditions) were almost consistently associated with HCU, which confirmed and extended the cross-sectional positive associations between need factors and HCU [5]. This knowledge may assist in managing HCU. Since most of the studies were conducted in North America or Europe, future longitudinal studies from other regions are urgently required.

## Figures and Tables

**Table 1:** Search strategy for Medline.

#1	Health care
#2	Health service *
#3	#1 OR #2
#4	Use
#5	Utili *
#6	#4 OR #5
#7	#3 AND #6
#8	GP visits
#9	Hospital admission
#10	Hospitalization
#11	Specialist visits
#12	Doctor visits
#13	Physician visits
#14	General Practitioner visits
#15	#7 OR #8 OR #9 OR #10 OR #11 OR #12 OR #13 OR #14
#16	Andersen model

Note: The asterisk (*) is a truncation symbol. The number sign (#) refers to the search order.

**Table 2:** Key characteristics and main findings of studies included in the final synthesis (*n* = 10).

First Author	Country	Assessment of Health Care Utilization	Waves and Duration	Sample Description	Sample Size; Age; Females in Total Sample	Results: Predisposing Factors	Results: Enabling Factors	Results: Need Factors	Results: Psychosocial Factors
Al Snih (2006) [12]	United States	Number of physician visits and hospitalizations during the last twelve months	Two waves from 1993 to 1996	Hispanic Established Population from the Epidemiological Study of the Elderly	*n* = 1987 M: 72.6 SD: 6.1 ≥65 59.5%	According to multiple regression analysis, age (ß = 0.04, *p* < 0.05) and being female (ß = 0.97, *p* < 0.0001) were related to physician visits. Marital status, education and nativity remained insignificant.	Receiving Medicare only (ß = 0.89, *p* < 0.05) or Medicare and Medicaid (ß = 1.33, *p* < 0.001) was significantly associated with physician visits. Number of children, financial strain and having a usual source of care were not significant predictors.	Some medical conditions, such as diabetes, were significantly correlated with both physician visits (ß = 1.10, *p* < 0.0001) and number of hospitalizations (ß = 0.94, *p* < 0.001), as well as the number of medications (physician: ß = 0.65, *p* < 0.0001, hospital: ß = 0.33, *p* < 0.0001). Having a limitation in the activities of daily life was related to hospitalizations (ß = 2.74, *p* < 0.0001). Cognitive impairment and depressive symptoms remained insignificant.	Not applicable
Clay (2011) [13]	United States	Time since the last nonsurgical overnight hospital admission	Nine waves from 1999 to 2005	Community-dwelling adults aged 65 years and older	*n* = 942 M: 75.3 SD: 6.7 65–106 50.7%	Univariate Cox proportional hazard ratios show that race (African American vs. Caucasian: OR: 0.74, 95% CI: 0.59–0.93) and age (OR: 1.03, 95% CI: 1.01–1.05) were significantly related to the outcome variable. Gender, marital status, education and residence were not.	Social support (OR: 1.04, 95% CI: 1.00–1.09) and perceived discrimination (OR: 0.88, 95% CI: 0.77–0.99) were significantly correlated with the time gap. Mental state and private insurance were not.	Physical health (OR: 0.97, 95% CI: 0.96–0.98), limitations among activities of daily life (OR: 1.19, 95% CI: 1.10–1.29) and physical performance (OR: 0.91, 95% CI: 0.89–0.95) were significantly associated with the time since the last nonsurgical overnight hospital admission.Depressive symptoms (OR: 1.09, 95% CI: 1.04–1.14), anxiety (OR: 0.96, 95% CI: 0.93–0.98) and mental health (OR: 0.98, 95% CI: 0.97–0.99) were significantly correlated with the time gap.	Not applicable
Gabet (2019) [14]	Canada	Having used an emergency department during the last twelve months	Two waves from 2017 to 2018	Homeless people from Montreal	*n* = 270 18–39: 5.2% 40–49: 38.2% ≥50: 56.6% 42.2%	Not applicable	According to multiple logistic regression, specialized ambulatory service use (OR: 1.74, 95% CI: 1.00–3.01) and stigma (OR: 0.70, 95% CI: 0.56–0.89) were significantly associated with emergency department use.	Substance use disorders (OR: 1.70, 95% CI: 1.01–2.87) and perceived physical health (OR: 0.75, 95% CI: 0.58–0.98) were significantly correlated with emergency department utilization.	Not applicable
Hadwiger (2019) [15]	Germany	Six or more physician consultations during the last three months	Seven waves from 2002 to 2014	German Socio-Economic-Panel	*n* = 28,574 M: 53.6 SD: 16.7 17–102 55.6%	The regression results show that being a frequent attender was significantly associated with lower age (OR: 0.95, 95% CI: 0.94–0.96), having a partner (OR: 1.22, 95% CI: 1.07–1.41) and non-working (OR: 1.35, 95% CI: 1.22–1.50).	Logarithmized equivalent income and having a private health insurance remained insignificant.	Frequent attenders were likely to have a lower physical health (reversed OR: 1.11, 95% CI: 1.11–1.12) and mental health composite score (reversed OR: 1.05, 95% CI: 1.05–1.05).	Not applicable
Hajek (2017a) [19]	Germany	Number of physician visits during the last three months	Two waves from 2005 to 2010	German Socio-Economic-Panel	*n* = 11,310 M: 51.8 SD: 16.4 17–100 55.4%	According to Poisson regression, age, marital status, education and employment status were not significantly related to the number of physician visits.	The logarithmized equivalent income remained insignificant.	The number of physician visits was positively associated with decreased self-rated health (ß = 0.40, *p* < 0.001) and being severely disabled (ß = 0.18, *p* < 0.001).	An external locus of control was positively correlated with higher levels of physician visits (ß = 0.00, *p* < 0.05). Internal locus of control was not significant.
Hajek (2017b) [16]	Germany	Number of GP visits, specialist visits and having had a hospital stay during the last twelve months	Two waves from 2008 to 2011	German Ageing Survey	*n* = 1372 M: 64.3 SD: 11.2 40–95 52.2%	Regarding fixed-effects regression, being retired (ß = 0.17, *p* < 0.05) or not employed (ß = 0.18, *p* < 0.05) was related to more physician visits. A higher age was associated with a having a hospital stay (OR: 0.91, 95% CI: 0.84–0.98), as well as not being employed (OR: 2.37, 95% CI: 1.01–5.56). Marital status remained insignificant.	Logarithmized equivalent income and self-rated accessibility of doctors were not significant predictors.	Self-rated health was associated with all GP visits (ß = 0.11, *p* < 0.001), specialist visits (ß = 0.20, *p* < 0.001) and a hospital stay (OR: 1.77, OR: 1.34–2.32). The number of chronic diseases was related to more GP visits (ß = 0.04, *p* < 0.01) and specialist visits (ß = 0.06, *p* < 0.01). Overweight (ß = −0.16, *p* < 0.05) and obesity (ß = 0.24, *p* < 0.05) were related to a lower number of specialist visits. Underweight, currently smoking and physical activity remained insignificant.	Not applicable
Hajek (2018) [18]	Germany	Number of GP visits and specialist visits during the last three months	Two waves during a ten-month period	AgeQualiDe	*n* = 861 M: 89.0 SD: 2.9 85–100 69.0%	Poisson fixed-effects regression did not detect age or marital status as significant correlates.	Social network was not significantly correlated with GP visits.	Increasing cognitive impairment (ß = 0.17, *p* < 0.05) and increasing depressive symptoms (ß = 0.04, *p* < 0.1) were significantly related to GP visits, while functional impairment and the number of chronic conditions were not.	Not applicable
Hajek (2020) [17]	Germany	Having had a hospital visit during the last six months	Two waves during a ten-month period	AgeQualiDe	*n* = 861 M: 89.0 SD: 2.9 85–100 69.0%	According to random-effects regression, age, sex and marital status were not associated with hospitalization.	A higher social network (OR: 1.15, 95% CI: 1.06–1.25) was associated with a higher likelihood of hospitalization. Education remained insignificant.	A higher number of chronic conditions (OR: 1.06, 95% CI: 1.02–1.10) and increased depressive symptoms (OR: 1.11, 95% CI: 1.05–1.18) were significantly related to hospitalization. Moreover, the interaction between social network and functioning (OR: 0.98, 95% CI: 0.97–0.99) was associated with hospitalization.Cognitive impairment and functioning were not.	Not applicable
Kim (2016) [20]	South Korea	Any outpatient health services utilization during the last twelve months	Two waves from 2010 to 2012	Korea Health Panel	*n* = 11,362 M: 51.1 SD: 17.8 57.1%	Respecting logistic regression, outpatient health services utilization was related to being female (OR: 3.12, *p* < 0.1), age (OR: 0.95, *p* < 0.05) and being married (OR: 8.3, *p* < 0.05).	Education, household income, economic activity and insurance were not related to outpatient health services use.	Having a chronic disease was correlated with service utilization (OR: 2.81, *p* < 0.05), but not with disability.	Not applicable
Stein (2000) [21]	United States	Having had a hospital visit or an outpatient visit during the last twelve months	Two waves from 1990 to 1991	Homeless people living in Los Angeles County	*n* = 363 M: 38.1 18–70 30.0%	According to the pathway model, hospitalizations were significantly related to education (ß = −0.10, *p* < 0.05), being African American (ß = 0.09, *p* < 0.05) and drug use (ß = 0.13, *p* < 0.05). Ambulatory office visits were associated with alcohol problems (ß = −0.10, *p* < 0.05) and drug use (ß = 0.18, *p* < 0.01). Poor housing remained insignificant.	Having a place to go for health care was related to increased levels of ambulatory office visits (ß = 0.32, *p* < 0.001) and community support (ß = 0.10, *p* < 0.05). Hospitalizations were related to community support (ß = 0.10, *p* < 0.05) and barriers (ß = 0.17, *p* < 0.001). Health insurance and social support were not significant predictors.	Having a poor health was related both to ambulatory office visits (ß = 0.09, *p* < 0.05) and hospitalizations (ß = 0.12, *p* < 0.05). Psychotics and depression remained insignificant.	Not applicable

**Table 3:** Quality assessment of studies included in the systematic review.

First Author (year)	Study Objective	Inclusion and Exclusion Criteria	HCU Description	Comparison Group or Disorder-Specific HCU	Data Source	Missing Data	Statistics	Consideration of Confounders	Sensitivity Analysis	Sample Size (Subgroup)	Demographics	Results Discussed with Respect to Other Studies	Results Discussed Regarding Generalizability	Limitations	Conclusion Supported by Data	Conflict of Interest/Funders	% of Criteria Fulfilled by Study
Al Snih (2006)	✓	✓	✓	✓	✓	x	✓	✓	x	✓	✓	✓	✓	✓	✓	✓	87.5
Clay (2011)	✓	✓	✓	✓	✓	✓	✓	✓	x	✓	✓	✓	✓	✓	✓	✓	93.8
Gabet (2019)	✓	✓	✓	✓	✓	✓	✓	✓	x	✓	✓	✓	✓	✓	✓	✓	93.8
Hadwiger (2019)	✓	✓	✓	✓	✓	x	✓	✓	✓	✓	✓	✓	✓	✓	✓	✓	93.8
Hajek (2017a)	✓	✓	✓	✓	✓	x	✓	✓	✓	✓	✓	✓	✓	✓	✓	✓	93.8
Hajek (2017b)	✓	✓	✓	✓	✓	x	✓	✓	✓	✓	✓	✓	✓	✓	✓	✓	93.8
Hajek (2018)	✓	✓	✓	✓	✓	x	✓	✓	✓	✓	✓	✓	✓	✓	✓	✓	93.8
Hajek (2020)	✓	✓	✓	✓	✓	x	✓	✓	✓	✓	✓	✓	✓	✓	✓	✓	93.8
Kim (2016)	✓	✓	✓	✓	✓	x	✓	✓	x	✓	✓	✓	✓	✓	✓	✓	87.5
Stein (2000)	✓	✓	✓	✓	✓	✓	✓	✓	x	✓	✓	✓	✓	✓	✓	✓	93.8
% of criteria fulfilled by studies	100	100	100	100	100	30	100	100	50	100	100	100	100	100	100	100	92.5

**Notes:** x = not fulfilled; ✓ = fulfilled.

## Data Availability

Not applicable.
